# Basics for Improved Use of Phages for Therapy

**DOI:** 10.3390/antibiotics10060723

**Published:** 2021-06-16

**Authors:** Philip Serwer, Elena T. Wright, Jorge De La Chapa, Cara B. Gonzales

**Affiliations:** 1 Department of Biochemistry and Structural Biology, The University of Texas Health Center, San Antonio, TX 78229-3900, USA; wrighte@uthscsa.edu; 2 Department of Comprehensive Dentistry, The University of Texas Health Center, San Antonio, TX 78229-3900, USA; delachapaj@uthscsa.edu (J.D.L.C.); gonzalesc5@uthscsa.edu (C.B.G.)

**Keywords:** bacteriophage, double-stranded DNA, drug delivery vehicle, infectious disease, multidrug-resistant bacteria, murine model, native gel electrophoresis, phage therapy, bypassing limitations of

## Abstract

Blood-borne therapeutic phages and phage capsids increasingly reach therapeutic targets as they acquire more persistence, i.e., become more resistant to non-targeted removal from blood. Pathogenic bacteria are targets during classical phage therapy. Metastatic tumors are potential future targets, during use of drug delivery vehicles (DDVs) that are phage derived. Phage therapy has, to date, only sometimes been successful. One cause of failure is low phage persistence. A three-step strategy for increasing persistence is to increase (1) the speed of lytic phage isolation, (2) the diversity of phages isolated, and (3) the effectiveness and speed of screening phages for high persistence. The importance of high persistence-screening is illustrated by our finding here of persistence dramatically higher for coliphage T3 than for its relative, coliphage T7, in murine blood. Coliphage T4 is more persistent, long-term than T3. *Pseudomonas chlororaphis* phage 201phi2-1 has relatively low persistence. These data are obtained with phages co-inoculated and separately assayed. In addition, highly persistent phage T3 undergoes dispersal to several murine organs and displays tumor tropism in epithelial tissue (xenografted human oral squamous cell carcinoma). Dispersal is an asset for phage therapy, but a liability for phage-based DDVs. We propose increased focus on phage persistence—and dispersal—screening.

## 1. Introduction

Success of attempts to engineer phages for phage-based therapies [1,2] has constraints based on the following: phage-host, phage-environment, and host-environment interactions. The environment has multiple components and can vary. Thus, multiple systems are involved, and the engineering required is, of necessity, systems engineering. This is the case for phage therapy of bacterial disease (reviews [3,4,5]) and for phage-based delivery of both antigens (reviews [6,7]) and drugs (reviews [8,9]). For example, use of phages for these purposes has improved outcome as non-targeted, patient activated removal of phages from blood decreases (persistence increases). High phage persistence should be a high priority, as high persistence is one of the functions essential for efficacy and, in some cases, persistence may be efficacy limiting.

In the past, high persistence has sometimes been considered [10,11,12], but has been surprisingly low in priority during work on phage therapy of bacterial disease (reviews [1,3,4,5,13]). Persistence was not considered during the therapy-optimization of several phages, including phage T7 [9,14,15,16], by use of genetically engineered additions. 

In addition, when serially propagated, particularly in the wild, recombinant phages and other viruses undergo spontaneous genome re-engineering by mutation/selection [17,18]. The apparent reason is distortion of the original engineering by an inserted gene. Thus, without systems engineering-generated genomic stability, phages will likely either lose an added function or become otherwise weakened for their therapeutic purposes. 

To maximize persistence and provide genomic stability, an advantage exists to obtaining needed functions via phage isolation from the environment (de novo phage isolation). With this strategy, genomic stability is already systems engineered by mutation/selection in the environment. At the current stage of knowledge, this engineering is, at the least, a good hedge against inadequate human engineering. It is also possibly the only current way to be successful in improving phage-based therapies.

Optimizing use of de novo phage isolation implies increasing the following three aspects: (1) the rate of phage isolation from the environment, (2) the diversity of the phages isolated, and (3) the rate at which screening for required phage characteristics is achieved. The assumption is that phages with required characteristics, such as high persistence, will eventually be found. Here, we discuss past developments to increase the first two aspects. We present data that project productivity of future increasing of the third aspect, i.e., rate of screening. We also present data that indicate the use of phage-based drug delivery vehicles (DDVs) has potential for tumor-selectivity, even if wild type phages are used.

## 2. Increasing the Rate and Diversity of Lytic Phage Isolation

### 2.1. Some Basics

Increasing the isolation rate and the diversity of phages isolated has previously been achieved via isolation and propagation in gelled media without liquid culture involvement. The gel used was made of dilute agarose, usually 0.10–0.2% [19,20,21,22]. Nonetheless, in-dilute gel phage isolation has not been widely used. The more typical isolation procedures have one or more of the following components: (1) phage enrichment via propagation in liquid culture (liquid enrichment culture), (2) filtration to remove bacteria and other micron-sized or larger objects, (3) treatment with chloroform to lyse bacteria, and (4) plating on traditional agar gels (0.7–1.0%) (reviews: [22,23]). Each one of these procedural aspects (i.e., 1–4) selects against phages of types known to have members. Most dramatically, (2) and (4) eliminate relatively large phages [19,20,21,24]. Membrane enveloped phages are also under-sampled [25].

Not only does the in-dilute gel strategy encompass isolation of phages of all known types, this strategy can also result in phage isolations at a rate 10–100× as high as more traditional procedures. The limit to the isolation rate is capacity for naming, cataloguing, and storing the phages isolated. Isolation rates of 54 phages per week per person have been achieved (Serwer laboratory, unpublished work) and can presumably be raised by further advancing procedures.

### 2.2. History

In-dilute gel phage isolation strategy was stimulated by work with phage G, which is the isolated phage with the longest genome. Physically, the G genome is 626 kb long [26]. Phage G was originally isolated in Italy [27] as the result of unusual circumstances [21,27]. The double-stranded DNA genome of phage G was subsequently used for calibrating DNA fractionations by both rate zonal centrifugation and pulsed field gel electrophoresis [28]. However, at least in the Serwer laboratory, phage G propagations via liquid culture were sub-par, to the point that one sometimes ended with fewer phages than inoculated. Finally, we achieved an acceptable liquid culture lysate, and decided to purify the phages by buoyant density centrifugation in a cesium chloride density gradient. This resulted in the production of expelled phage DNA, the phages having been destroyed.

The in-gel propagation initiative was, in part, a response to this “last straw” sequence of events. Several investigators started minimizing assumptions. This process has continued with the collaborative finding that (1) the bacterial host for phage G is not the relatively large *Bacillus megaterium* initially proposed [27], but an average-sized *Lysinibacillus* [29], and (2) anecdotally, phage G is not well preserved by freezing. In the Serwer laboratory’s experience, centrifugation in a sucrose gradient has been, so far, the least damaging to large environmental phages, including phage G [24,30]. To illustrate the benefits of the above information, the phage G preparation used for a 6.1 Å cryo-EM structure of the DNA-enclosing outer shell [29] started as a two-Petri plate, top-agarose lysate (plate stock) and ended with purification via rate zonal centrifugation in a sucrose gradient [24].

The following question arises: given the above, do we have reason to believe that current in-gel procedures are capable of isolating bacteriolytic, propagating agents of all existing types? The answer is no. First, in-gel, bacteriolytic, propagating agents exist that, structurally, do not resemble known phages [31]. We do not know whether a spectrum of these agents exists with, perhaps, known phages at one end of the spectrum. Second, we have seen plaques of bacteriolytic agents that do not further propagate, as though accidental, uncontrolled characteristics of the supporting gel are necessary for propagation. Third, phages of several known types have eukaryotic virus homologs. For example, double-stranded DNA phages are homologs of herpesviruses [32,33]. Phages in other categories also have eukaryotic virus homologs [34,35]. However, to our knowledge, no coronavirus-like phage has ever been described in a publication. This inconsistency is possibly explained by the data that indicate that long tails assist large phage propagation [24] and that coronaviruses are as large, but without tails. Good reason exists to try to isolate “corona-phages” with in-dilute gel techniques [36].

## 3. The Persistence Factor

### 3.1. Needed Phage Characteristics

Increasing the number and diversity of phages isolated is precursor to screening the phages for needed characteristics. Positive screening to obtain lytic phages is at the top of the priority list for phage therapy. This screening is initially done via positive selection for clear plaques [22,23]. A positive clear plaque-screen may be accompanied by negative genomic sequencing-screens for genes that encode for lysogeny, toxins, and antibiotic resistance genes [22,23]. These screens are not further discussed here.

However, screening for lytic phages is not always sufficient, as illustrated by the following recent success/failure pair. Lytic phages for *Acinetobacter baumannii* were used to reverse a systemic, multi-drug resistant human infection that appeared certain to be fatal before the phage therapy [37]. This case caught the imagination of the public [38,39], although it was similar to cases of the phage therapy of typhoid fever from ~80-years earlier [40,41]. Nonetheless, a subsequent attempt to repeat this performance failed [42]. The authors of [42] included four of the authors of [37]. In [42], phage titer vs. time in blood was determined. The result was that five minutes in blood caused more than a 95% reduction in phage titer in systemic circulation.

Although this (low) phage persistence was possibly not the only cause of the failure, it was likely to be a major contributor for the following reason. The most rapidly propagating phages, T3 and T7, produce a burst after 13–15 min at 37 °C in aerated laboratory culture. A burst of 100 phages per bacterium is typical in liquid culture. In a person, (asynchronous) phage production is likely to be fewer than the implied ~33 phages per 5 min for T3 and T7 in liquid culture. If it is fewer than 20 phages per 5 min, then, the titer vs. time in blood of [42] would imply that phages will be removed from blood more rapidly than they are being produced. That is to say, in this case, phage persistence would have been too low for therapy to be effective for a bacteremia. To be optimally effective, the phages used for therapy should multiply as rapidly as possible, and should be as highly persistent as possible, until an upper limit most likely determined by toxicity [43,44] of lysis-produced bacterial endotoxin. We note that the reasons for low persistence are not knowable without additional data, and might include high dispersion.

### 3.2. Phage Persistence in Blood

If the assumption is made that the low phage persistence in reference [42] is universal, then phage therapy would likely be as hopeless as previously stated ([45]; review [46]) and found: *Streptococcus* [47], *Staphylococcus* [48]. A reason for the failures was found to be loss of phages induced by blood, pus, acites fluid, and urine [47]. Together, these data imply low persistence. However, as suggested by others in the past [3,48], persistence level cannot be assumed, and should be determined during screening of environmental phages.

Indeed, the literature of the distant past contains examples of successful phage therapy accompanied by higher phage persistence. For example, intraperitoneal (IP) inoculation of an anti-*Shigella dysenteriae* phage into white mice results in persistence of approximately 2 h. When these mice are intracerebrally infected with *Shigella dysenteriae*, IP inoculated phages cross the blood/brain barrier to multiply in the brain and, sometimes, cure the infection [49]. In addition, the effectiveness of various phages against *Salmonella typhimurium* is in support of the idea that dramatic differences are linked to differences in the phage used for typhoid fever both in a murine model [50,51,52] and in people [40,41]. More recently, cell culture-based studies have confirmed relatively high persistence of some phages, and found polar crossing by phages of cell membranes into and out of cells (sometimes called transcytosis). Transcytosis is proposed to be a human health-promoting process [53,54].

At one extreme in the spectrum of phage persistence, as measured via blood titer, we found persistence surprisingly high for phage T3. The peak blood titer of phage T3, after IP injection into a mouse, did not decrease by more than 2× until 3–4 h after injection [55]. In this case, even if average phage production was as low as 2.5 phages per 5 min per bacterial cell and the initial bacterial concentration was 10^7^ per mL (approaching lethal levels), then phages would, conservatively, gain the upper hand within 3 h, assuming a bacterial doubling time of 1 h and 0.001 for the multiplicity of infection. Phage production could be even lower and still yield success.

Other studies [12] find that the T3 relative, T7, is less persistent. The blood titer of T7 is more than 90% removed by 1.0 h after IP inoculation. However, the possibility of murine host-specific effects causes uncertainty that the T3/T7 difference is phage-, and not murine host-, determined.

## 4. More Rigorous Comparison of Persistence: Phage T3 vs. Phage T7

**Figure 1:**
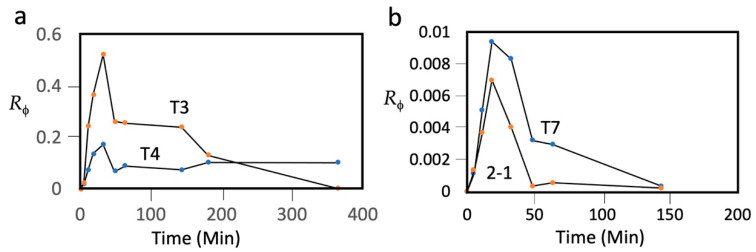
*R*_ϕ_ vs. time for phages (**a**) T3 and T4; and (**b**) T7 and 201phi2-1 (indicated by 2-1). The hosts for plaque counts were the following (phage, followed by host): **T3**, *E. coli* IJ511/F’lac (F42) [46]; **T4**, *E. coli* K-12 HfrC (PO2A) tonA22 garB10 ompF627 (T2-R) relA1 pit-10 spoT1 fadL? phoA4(Am) phoM510 mcrB rrnB2 (λ); **T7**, E. coli BB/1/3; and **201phi2-1**, *P. chlororaphis*. The temperatures of incubation and plaque supporting gels were the following: **T3**, 37 °C, 3.5 h.; **T4**, 37 °C, 6 h.; **T7**, 37 °C, 4.5 h.; and **201phi2-1**, 30°C, 18 h. The media used for the plaque-supporting gel were **T3**, 0.5% agar in T broth (1% tryptone, 0.5% NaCl); **T4**, 0.5% agar in T broth; **T7**, 0.5% agar in T broth; and **201phi2-1**, 0.18% Seakem Gold agarose (Lonza) in 1.0% tryptone, 0.5% KCl, supplemented post-autoclaving with 0.002 M CaCl_2_. The hosts for T4 and 201phi2-1 do not plate any of the other phages in these conditions. The hosts for T3 and T7 plate T4 (only) among the other phages. T4 plaques were eliminated from T3 and T7 counts by restricting incubation time, as described above, which yielded T3 and T7 plaques that were 2–4 mm and clear while T4 plaques were turbid and pinpoint, if visible at all. Titers in the 0.1 mL inoculum were the following: T3, 1.1 × 10^11^ PFU/mL; T4, 2.8 × 10^11^ PFU/mL; T7, 4.0 × 10^10^ PFU/mL; and 201phi2-1, 9.1 × 10^10^ PFU/mL.

To make this T3/T7 comparison dependent on phage only, we have repeated it with T3 and T7 that were co-inoculated into a single mouse in 0.1 mL of a T3/T7 mixture. The inoculation was IP into an eight-week-old, female C57/BL6 mouse. After inoculation, 1.0 μL samples of tail blood were taken, at the times indicated in Figure 1, and diluted into 1.0 mL of T7/G buffer: 0.5 M NaCl, 0.01 M Tris-Cl, pH 7.4, 0.001 M MgCl_2_, and 1 mg/mL gelatin. Infectivity titers (plaque-forming units (PFU) per mL of blood) for T3 were obtained on host, *Escherichia coli* IJ511/F’lac (F42), on which T7 does not form plaques [56]. Infectivity titers for T7 were obtained on a T3-resistant host isolated here, *Escherichia coli* BB/1/3. For each phage recovered from blood, the ratio (*R*_ϕ_) of titer to the number of phages inoculated was plotted vs. time.

These plots had the following three features (T3: plot labeled T3 in Figure 1a; T7: plot labeled T7 in Figure 1b): (1) At 0.0–18.3 min, *R*_ϕ_ increased for both phages, formed a peak, and subsequently decreased. The maximum *R*_ϕ_ at 18.3–32.6 min will be called *R*_ϕ__Μ._ (2) The *R*_ϕ__Μ_ for T3 was 56× higher than it was for T7. (3) At 48.4 min, the T7 titer had decreased by 2.6×, and then proceeded to further decrease by an order of magnitude at 143.5 min, while the T3 titer did not decrease by 2.6× until after 143.5 min. The first feature indicates that more T7 than T3 was lost before and during entry of the phage into blood. This difference is not likely to be a sieving effect, as T3 and T7 have capsids indistinguishable in size [57], 30 nm by cryo-electron microscopy (genomic dsDNA length: T7, 39.937 kb; T3, 38.208 kb) [58,59]. Both have a relatively short tail; most structural genes are homologs [58].

The third feature indicates that, in blood, whatever is removing/inactivating the phages is less effective for T3 than it is for T7. This difference confirms the previous conclusion, discussed above, based on data from two different mice and two different laboratories. The second and third features, above, indicate that phage T7 is significantly less persistent than phage T3.

## 5. Comparison with Phages T4 and 201phi2-1

We also included phages T4 and 201phi2-1 in the mixture used for T3 and T7 (i.e., a four-phage mixture). Phage T4, similar to phages T3 and T7, is a double-stranded DNA coliphage. T4 has a capsid with a DNA-containing cavity that has a volume about 4× larger than the volume of the T3 and T7 counterpart (T4 genome length = 172 kb) [60]. T4 has a relatively long (92.5 nm) tail [60]. Phage T4 is of interest due to its potential as a DDV, as indicated by (1) previous [61] dye-in-phage loading experiments, and (2) its use for transferring packaged proteins [62], surface antigens [6,63], and DNA [62] to eukaryotic cells.

The *R*_ϕ_ vs. time plot for T4 (plot labeled T4 in Figure 1a) was lower than, but had the shape of, the plot for T3 at times up until 143 min. Thus, T4 was more restricted than T3 during entry into blood. However, after 143 min, the T3 *R*_ϕ_ underwent a steep decrease, while the T4 did not decrease, even by 364 min. That is to say, after entry into blood, T4 was more persistent than T3 by over 3.7 h. The T4 *R*_ϕ_ did further decrease, by ~30×, when a sample was taken at 1391 min (not shown in Figure 1).

Phage 201phi2-1 is a double-stranded DNA phage with a capsid that contains a 316.674 kb dsDNA genome and with a 200 nm-long tail. The host is *Pseudomonas chlororaphis* [64]. Phage 201phi2-1 is of interest as it is found [64] to be a homolog of phage ϕKZ, a lytic phage that infects *Pseudomonas aeruginosa* [65,66], a major pathogen causing respiratory disease [67].

The *R*_ϕ_ vs. time plot for phage 201phi2-1 (plot labeled 2-1 in Figure 1b) revealed that 201phi2-1 was more restricted than all three other phages during entry into blood. Once in blood, the persistence of 201phi2-1 was also the lowest among the different phages, significantly lower than the persistence of T7.

## 6. Phage T3 Dispersion in a Tumor-Bearing Mouse

If high-persistence, wild type phages preferentially migrate to tumors after inoculation, then their potential as tumor-specific DDVs is increased. To our knowledge, there is only one test of wild type phage migration to tumors in the literature, and phage infectivity was found preferentially in the tumor. The publication date is 1940 [68] (review: [69]). The phage used is not characterized. In 1940, most characterizations routinely possible today were not yet achieved for any phage. The 1940 experiment is not yet, to our knowledge, repeated with a phage well-characterized by current standards. We chose to repeat this experiment with phage T3, as we had begun to test the possibility of using a T3 capsid as a DDV [70].

At 4.0 h after IP inoculation with 7.3 × 10^10^ PFU of phage T3 (in 0.1 mL), the various tissues of a tumor-bearing mouse were assayed for T3 plaque formers. The tumor had been generated by xenografting human Cal27 cells (oral squamous cell carcinoma) on the left flank of an athymic nude mouse, as previously described [71]. Although one inoculation was performed, two tumors were generated, one relatively large (~1.1 cc) and one too small to reliably assay for phage (~0.1 cc). The smaller tumor was mesial to the larger tumor and separated from it by 9 mm. The larger tumor was divided in two to assist homogenization for T3 phage detection. T3 was assayed in various organs after organ homogenization in T7/G buffer with a Dounce homogenizer. The blood T3 titer at the time of death was 3.3 × 10^7^ PFU/mL.

T3 was found to accumulate primarily in spleen, liver, lungs, brain, and heart, as seen via the T3 titer per gram of tissue (Table 1). The titer per gram is calculated in two ways. In column B, the titer per gram is presented without correcting for the contribution of the blood in each organ. In column C, the titer per gram is calculated after correction for the blood-associated titer. The blood-associated titer was calculated assuming fractional blood volumes from [72] for the various tissues and 3.3 × 10^7^ PFU/mL in blood. This latter titer implies significantly more loss of blood titer than seen at 4 h in the non-tumor-bearing mouse of Figure 1. The titers in spleen, liver, lungs, and brain were high enough so correction for blood-associated titer did not have a significant effect.

The larger tumor also accumulated T3 PFU, although not to the extent of the spleen, liver, lungs, and heart. Selectivity of the accumulation in the tumor is seen via the lower PFU per gram in two samples of the non-tumor-bearing skin (Table 1).

Accumulation in the liver, spleen, lungs, and even the brain, is an expected consequence of activity of the mononuclear phagocyte innate immune system (review [73,74]). Given the relatively high persistence of T3, the expectation is that the accumulation of T3 in the spleen and liver is low in comparison to what would occur with other, less persistent phages. The relatively high titer in the brain is reminiscent of the study in [49].

The accumulation of T3 in a tumor is a favorable sign for use of a T3-based, tumor-specific DDV. Accumulation elsewhere is an unfavorable sign. This latter accumulation might be suppressed either by use of another phage, obtained via environmental phage-isolation/screening, or by use of another route of T3 inoculation. However, the lower blood titer relative to the 4 h time of Figure 1 might have been caused by selective phage inactivation in the tumor (see Section 8), which would mean approximately two orders of magnitude more phage particles (most inactive) in the tumor than seen via titer. Further work is needed to test this possibility.

## 7. Increasing Screening Efficiency: Moving toward the Ultimate Objective in Phage Therapy

The above measurements of persistence are time- and resource-consuming enough to search for a more efficient, proxy procedure. As important as a more efficient procedure is, we are currently at a very early stage of development. Given recent lessons in the area of microbial preparedness [75,76,77], we should be treating this as a top priority. Documented deaths in the US from multi-drug resistant bacteria have risen from 23,000 in 2013 to 35,000 in 2019 [78].

In an attempt to engineer a breakout, we note the following. The average electrical surface charge density (σ) of phage T3 is negative, and 1.28× the negative σ of T7 at a pH of 7.4 in 0.05 M phosphate buffer [57]. Relative values of σ were determined by extrapolation of mobilities during native gel electrophoresis (NGE) to a gel concentration of zero in a polysaccharide gel that had no detected electro-osmosis. The probability (not certainty) of high persistence might be an increasing function of the magnitude of a negative σ. For example, red blood cells are protected from interaction with both one another and other cells by the negative charge of the surface-located sialic acids of membrane-embedded glycophorins [79,80]. More detailed tests should be undertaken regarding the correlation of persistence with the magnitude of negative phage σ. This process can be simplified by the following versions of native gel electrophoretic analysis: (1) phage samples from single plaques, without any phage purification or concentration [55], and (2) two-dimensional NGE to obtain relative σ values in a single gel [81].

Details of tail structure might also help to determine persistence. For example, phage T7 adheres to agarose gels during NGE, while phage T3 does not. T7 adherence does not detectably occur in the absence of tail fibers [82], indicating that the tail fiber is the primary adherent phage component.

In this area of phage therapy, perfection is not necessary, as phage therapy is typically done with a mixture (cocktail) of phages. For the sake of illustration, imagine that (1) two separate 5-phage cocktails are made, one with and the other without persistence-screening, and (2) persistence screening modestly increases the probability per phage of high persistence, from 0.1 to 0.3. The probability that at least one phage has high persistence goes from 0.41, without persistence screening, to 0.83 with persistence screening. 

## 8. Moving toward the Ultimate Objective in Anti-Tumor Therapy

When developing an anti-tumor strategy, one wants to know that the strategy includes ways to manage the two success-blocking limitations that have arisen with all past anti-tumor drug therapy of metastatic cancer: cancer cell mutation to drug resistance [83,84], and drug toxicity to healthy cells [85,86,87]. If we have high persistence phage-based DDVs, then one limitation-bypassing strategy has the following tactical objectives: (1) zero DDV uptake into healthy tissue; (2) zero drug leakage from the DDV during circulation in blood (see [70]); (3) uptake, not necessarily rapid (possibly, 1–2 days), into tumors; and (4) rapid (i.e., a matter of minutes) drug release in tumors, which might be achievable via gates that are naturally on dsDNA phage capsids [70]. Of course, meeting these objectives is beyond our current capacity. However, working toward them will yield progressively more effective therapies. The partially tumor-selective distribution observed here for phage T3 provides a step in that direction. The central point is that, with high persistence and low leakage, a DDV could circulate for hours/days while slowly accumulating in tumors. Finally, the point has been made [70] that partial reaching of the above tactical objectives may be sufficient, as the probability of a toxicity-generating series of tandem DDV-associated events is the product of the probabilities of each of the events.

If, in contrast to the above, a strategy does not have a systematic way to eliminate past success-blocking limitations, then skepticism is a natural response. A corollary is skepticism towards any strategy based on finding a single novel biochemical target that is not subject to the success-blocking limitations of the past. Frustrated efforts have been so numerous that one may assume that these efforts are doomed to either failure or weak results before they begin.

If use of the above DDV-based strategy leads to the accomplishment of its objectives, then the major limitation would become cancer cell evolution to either pump the anti-tumor drug out of cells [85,86,87], or possibly to inactivate the drug. Thinking ahead on this critical point leads to the following conclusion: if a DDV-based strategy sufficiently protects healthy cells from the drug, then this strategy can be used to raise the levels of tumor-delivered drugs and/or drug potency per molecule for an anti-tumor drug. This aspect would be used to destroy tumor cells before they evolve to either remove or destroy the drug.

The most difficult tactical objective is zero uptake into healthy tissue. Thinking ahead on this point leads to the following conclusion: in a healthy person, blood is largely not accessible to intestinal bacteria [88], even considering the possibility that blood is not 100% sterile [89]. In contrast, the high rate of blood vessel-entry of phages, as illustrated in Figure 1, is reasonably imagined to arise from the smaller-than-bacteria size of phages. Perhaps the finding that the T4 *R*_ϕM_ is lower than the T3 *R*_ϕM_ (Figure 1a), even though T4 is more persistent at later times, is caused by T4’s larger size-induced, slower epithelium-permeation. Slower epithelium permeation also can explain the slight rise in T4 titer after 143.5 min. This possibility advocates testing of prevention of phage particles migrating to healthy tissue via the use of even larger phages and phage capsids as anti-cancer DDVs.

If DDVs can be used to keep anti-cancer drugs away from the interior of healthy cells, then selective permeation of tumors by DDV-associated drugs should be achievable. This is due to the fact that the blood vessels of tumors, but not healthy tissues, have pores that allow selective access to tumors. Tumors also have relatively poor lymphatic systems to remove DDVs. Together, these two effects are called the enhanced permeability and retention (EPR) effect [90,91,92]. Variability of tumor access via the EPR effect [90,91,92] is projected to be best managed with a high-persistence DDV.

## 9. Conclusions and a General Perspective

The strategic ignoring of persistence has led to attempts to improve phage therapy with phages of sub-optimal persistence, for example, phage T7 (above). The data presented in Figure 1 indicate that (1) T7 is not a good candidate for phage therapy, with or without persistence-independent genetic modifications, and (2) the T7 relative, T3, is a much better choice. In our opinion, strategic deficiencies of this type cause the skepticism towards and the slowness in developing phage therapy of infectious diseases.

If high persistence becomes a feature of the environmental phages (or other viruses) selected for therapies, the perspective improves for both phage/virus DDV-enhanced anti-cancer therapy and phage therapy of multidrug-resistant bacterial disease. The screening-based strategy proposed here is aligned with previous high intensity screens used to produce “magic bullets” whose mechanisms were subsequently learned. These “magic bullets” include several antibiotics [93,94] (recent comprehensive review [95]) and anti-cancer drugs [96,97,98].

## Figures and Tables

**Table 1:** Distribution of phage T3 at 4.0 h after inoculation into a mouse that was bearing a xenografted oral squamous cell carcinoma tumor. Following euthanization by isofluorane inhalation and confirmation by cervical dislocation, organs were surgically excised and weighed. Phages in each organ were then released and dispersed with a Dounce homogenizer. The phages were titered on host, *E. coli* BB/1, after dilution in T7/G buffer.

A. Tissue	B. PFU/g × 10^7^	C. PFU/g × 10^7^
Liver	29.8	29.1
Spleen	123	123
Lung	53.8	53.5
Brain	9.7	9.6
Heart	45.3	-
Tumor	17.6	17.5
Tumor	8.1	8.0
Skin	0.18	0.062
Skin	0.31	0.19

## Data Availability

The data presented in this study are all available within one or more of the following: Figure, Table and Text.
